# *Vibrio owensii* Induces the Tissue Loss Disease *Montipora* White Syndrome in the Hawaiian Reef Coral *Montipora capitata*

**DOI:** 10.1371/journal.pone.0046717

**Published:** 2012-10-08

**Authors:** Blake Ushijima, Ashley Smith, Greta S. Aeby, Sean M. Callahan

**Affiliations:** 1 Department of Microbiology, University of Hawaii, Honolulu, Hawaii, United States of America; 2 Hawaii Institute of Marine Biology, Kaneohe, Hawaii, United States of America; University of New South Wales, Australia

## Abstract

Incidences of coral disease in the Indo-Pacific are increasing at an alarming rate. In particular, *Montipora* white syndrome, a tissue-loss disease found on corals throughout the Hawaiian archipelago, has the potential to degrade Hawaii’s reefs. To identify the etiologic agent of *Montipora* white syndrome, bacteria were isolated from a diseased fragment of *Montipora capitata* and used in a screen for virulent strains. A single isolate, designated strain OCN002, recreated disease signs in 53% of coral fragments in laboratory infection trials when added to a final concentration of 10^7^ cells/ml of seawater. In addition to displaying similar signs of disease, diseased coral fragments from the field and those from infection trials both had a dramatic increase in the abundance of associated culturable bacteria, with those of the genus *Vibiro* well represented. Bacteria isolated from diseased fragments used in infection trails were shown to be descendants of the original OCN002 inocula based on both the presence of a plasmid introduced to genetically tag the strain and the sequence of a region of the OCN002 genome. In contrast, OCN002 was not re-isolated from fragments that were exposed to the strain but did not develop tissue loss. Sequencing of the *rrsH* gene, metabolic characterization, as well as multilocus sequence analysis indicated that OCN002 is a strain of the recently described species *Vibrio owensii*. This investigation of *Montipora* white syndrome recognizes *V. owensii* OCN002 as the first bacterial coral pathogen identified from Hawaii’s reefs and expands the range of bacteria known to cause disease in corals.

## Introduction

Coral disease is a progressing threat to many reefs around the world. Reefs in the Florida Keys and Caribbean have been devastated since the first accounts of coral disease were documented in the early 1970s [1]–[5], and reports of disease in the Indo-Pacific are increasing [6]–[12]. Major disease outbreaks can lead to breakdown of reef structure, disruption of local ecosystems, and threaten tourism-dependent economies of areas like Hawaii and the Caribbean [8], [13]–[15]. Understanding coral disease is critical in developing management and conservation strategies to protect these valuable resources. However, disease processes are complex and our knowledge of causal factors of disease is extremely limited [5], [16].

Bacteria have been implicated as the etiological agent for several coral diseases [17]–[26]. The coliform bacterium *Serratia marcescens*, an opportunistic human pathogen commonly found in sewage, causes acroporid serratiosis in the Elkhorn coral *Acropora palmata.* [19], [27], [28]. *Vibrio shiloi*, an intracellular coral pathogen that attacks zooxanthellae following penetration of coral cells, causes bleaching [29]–[31]. *Vibrio coralliilyticus* causes cell lysis of *Pocillopora damicornis* at elevated temperatures or bleaching at lower temperatures [20], [32]. *Vibrio coralliilyticus* has also been implicated in outbreaks of tissue loss diseases (white syndromes) in corals in Palua, Marshall Islands, and the Great Barrier Reef [25], [33].

**Figure 1:**
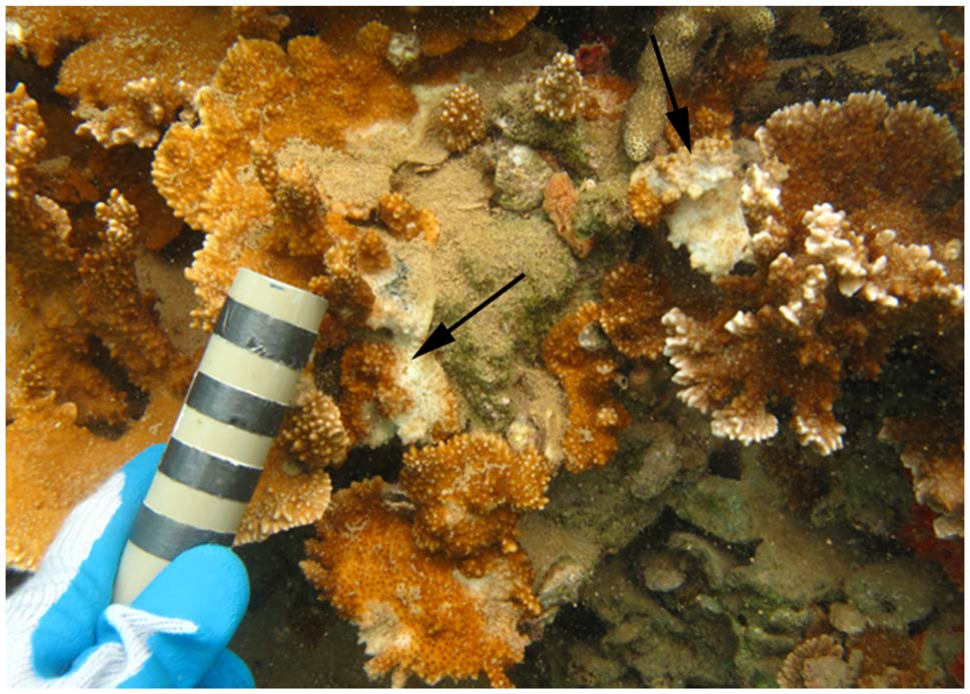
Subacute tissue loss pattern of cMWS. *M. capitata* colony from the field with disease signs of chronic *Montipora* white syndrome. The colony also shows algal overgrowth of a majority of the skeleton exposed by the infection. Arrows indicate progressing disease fronts. Bands on the scalar bar represent 1 cm increments.

In Hawaii, coral disease is an emerging problem. Twelve diseases have been described from across the archipelago and the frequency of disease outbreaks has increased [9], [34], [35]. *Montipora* white syndrome (MWS) is a disease of concern that has been reported across the archipelago [34], [35]. MWS is a chronic and progressive disease that produces a subacute tissue-loss pattern (Figure 1). There is a positive association between levels of MWS and *M. capitata* coverage, following the standard host-pathogen relationship [34]. MWS is transferred by direct contact between coral fragments, and there is no apparent link between MWS and time of year, suggesting that water temperature does not play a major role in the infection process [34]. Recently, the reefs of Kaneohe Bay experienced outbreaks of an acute tissue loss disease in *M. capitata* (Aeby, unpublished data). Therefore, we will refer to the slower moving MWS as chronic *Montipora* white syndrome (cMWS) to differentiate between the two types of lesions.

In this study a pathogen responsible for cMWS was isolated and classified as *Vibrio owensii* strain OCN002 based on *rrsH* gene sequence, multi-locus sequence analysis, colony morphology, and metabolic capabilities. Isolation of OCN002 in pure culture, the demonstration of virulence by exposure of healthy *M. capitata* fragments with an axenic culture, and the re-isolation of the introduced bacterium from coral showing signs of disease, fulfilled Henle-Koch’s 2^nd^, 3^rd^, and 4^th^ postulates [36], [37]. OCN002 expands the range of bacteria known to cause disease in corals and is the first bacterial coral pathogen identified in Hawaii.

## Results

### Initial Screen for a Potential cMWS Pathogen

**Figure 2:**
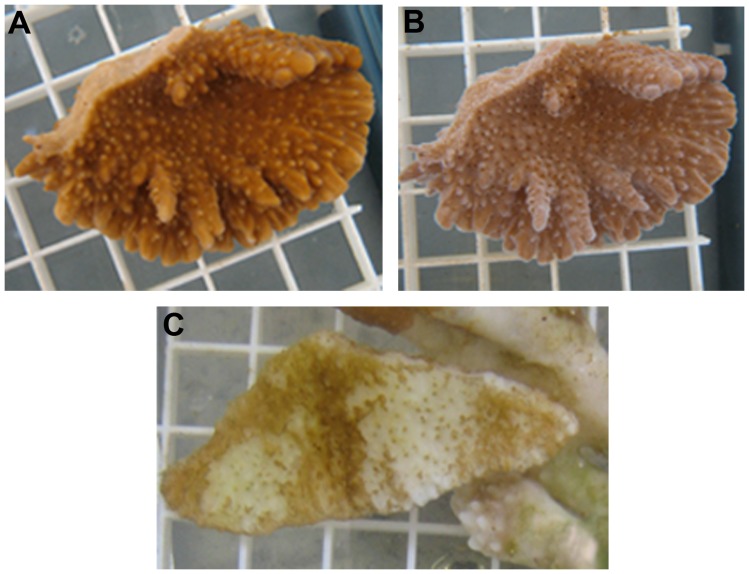
Progression of tissue loss in experimental coral fragment. (A) Coral pre-inoculation. (B) The same fragment exhibiting disease signs of cMWS 26 days post-inoculation as evidenced by exposed white skeletal elements. Fragments were processed for microbial analysis at this stage of tissue loss. (C) Experimental fragments with late stage cMWS. The majority of tissue has been lost exposing the white skeleton.

Contact-dependent transmissibility of MWS from diseased to healthy coral fragments suggested that the causative agent of MWS could be an infectious agent [34]. To isolate a potential pathogen, bacteria from a diseased coral fragment in Kāne

ohe Bay were plated on a complete medium, purified by streaking, and tested for their ability recreate signs of disease with healthy coral under laboratory conditions. A preliminary screen was conducted using corals inoculated with groups of 5 bacteria to rapidly assess the potential virulence of a large number of bacteria. Tissue loss only occurred in one of the 10 groups of isolates tested. All three of the coral fragments showed tissue loss after 21 days. The diffuse tissue loss, typical of cMWS, was characterized as the regression of coral tissue from the skeleton, resulting in exposure of white skeletal projections (Figure 2) and resembled corals with MWS observed in the field (Figure 1) [34]. None of the fragments in control aquaria or those exposed to other groups of bacteria showed signs of tissue loss.

To determine which bacterium in the group of five isolates was responsible for tissue loss, the five bacteria were tested individually for their ability to induce signs of disease. Corals exposed to four of the strains and those in control aquaria remained healthy. In contrast, the fifth strain caused tissue loss on all three coral fragments after 24 days. This strain was designated OCN002 and pursued further based on its potential as the etiologic agent of cMWS.

### OCN002 Induces Signs of cMWS

To test if strain OCN002 was responsible for the signs of disease in *M. capitata* observed above, healthy fragments of *M. capitata* were exposed to the putative disease-causing bacterial strain at a concentration of 10^7^ cells/ml, filtered seawater (FSW) as a control, or bacterial strain OCN004 as a second control. Strain OCN004 was isolated from extracellular mucus of healthy *M. capitata.* Seven of the 13 fragments exposed to OCN002 showed signs of disease after an average of 28 days subsequent to the addition of the bacteria (Figure 2B) (McNemar’s test, P = 0.014, n = 13/treatment). In contrast, none of the 13 fragments in control aquaria showed signs of tissue loss. The infection trials showed that inoculation of healthy coral with OCN002 causes tissue loss under laboratory conditions.

### Increased Bacterial Abundance Associated with Disease Signs in Infection Trials is Similar to that in Field Samples

**Figure 3:**
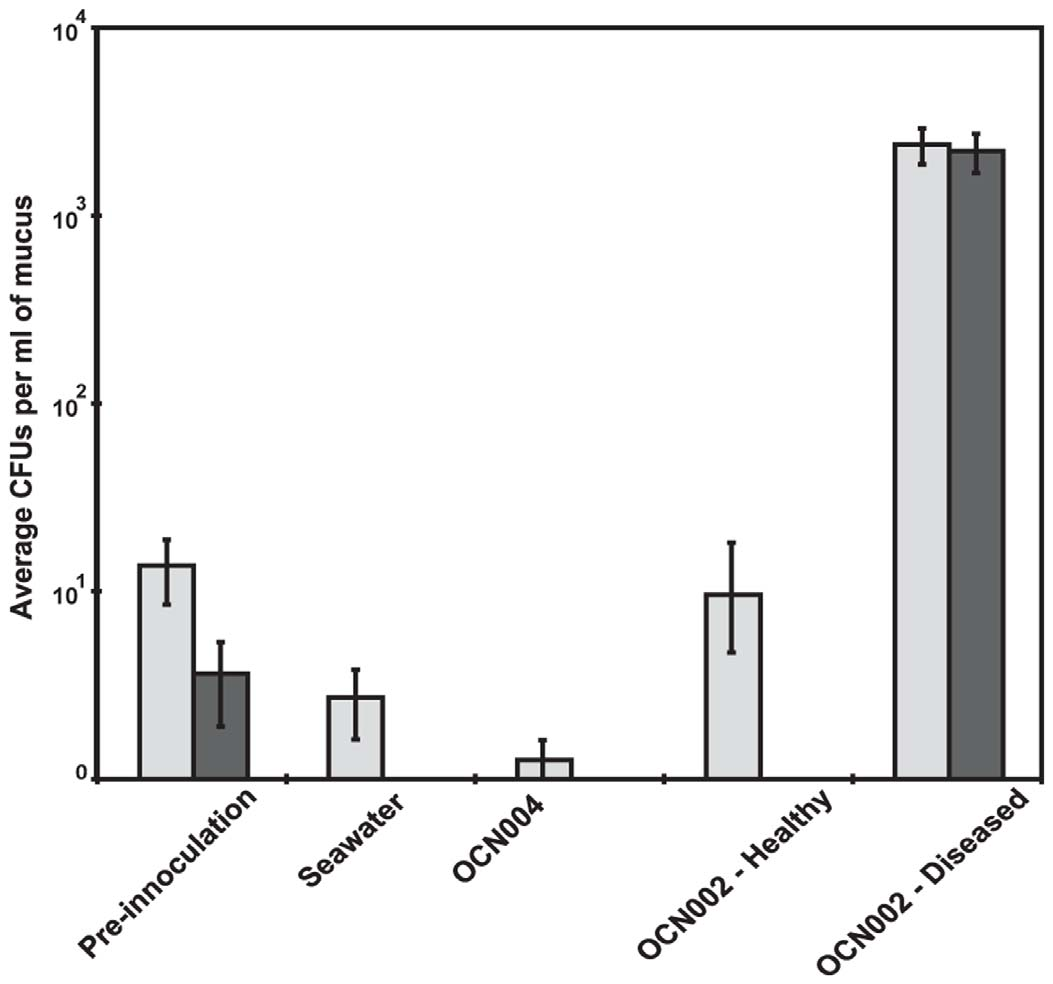
Differences in abundance of culturable bacteria among treatment groups from infection trials. Light and dark gray bars represent CFU per ml of coral mucus recovered on GASW (general marine medium) and TCBS (*Vibrio*-selective medium) solid media, respectively. ‘Pre-inoculation’ refers to CFU from mucus collected from representative coral fragments before infection trials; ‘Seawater’ refers to CFU from control fragments exposed to filtered seawater; ‘OCN004’ refers to CFU from fragments exposed to the control bacterium *Alteromonas* sp. OCN004; ‘OCN002-Healthy’ refers to CFU from fragments exposed to *V. owensii* str. OCN002 that remained healthy; ‘OCN002-Diseased’ refers to CFU from fragments exposed to OCN002 that developed tissue loss.

One of the hallmarks of coral disease is rapid over-growth of coral with bacteria not normally associated with healthy coral, with bacteria of the genus *Vibrio* well represented [22], [25]. A similar pattern of increased bacterial abundance and proportion of *Vibrio* spp. relative to those of healthy control fragments was found in diseased coral fragments in infection trials (Figure 3). Coral fragments in tanks inoculated with OCN002 that showed signs of disease had an average increase of 2.5×10^3^ (SE±9.55) CFU/ml, and a 78% (SE±26.35%) increase in *Vibrio* spp. (Table S2). In contrast, no significant increases in either bacterial abundance or proportion of *Vibrio* spp. were found in either group of control fragments (seawater or OCN004) nor from coral fragments exposed to the test strain (OCN002) that did not develop disease signs (Figure 3, Table S2). Coral fragments to which sterile seawater had been added had an average decrease of 1.0×10^1^ (SE±3.79) CFU/ml, and a 14% (SE±8.07%) decrease in *Vibrio* spp. proportions. Coral to which strain OCN004 had been added had an average decrease of 1.1×10^1^ (SE±4.52) CFU/ml, as well as a 16% (SE±7.72%) decrease in *Vibrio* spp. proportions (Table S2). Furthermore, the fragments in tanks inoculated with strain OCN002 that remained disease-free had an average increase of 1.3×10^1^ (SE±466.98) CFU/ml, but a 3% (SE±0.36%) decrease in the *Vibrio* spp. proportions (Table S2).

Similar to the laboratory results described above, mucus collected from healthy *M. capitata* directly from the field had a significantly lower abundance of culturable bacteria as compared to field collected samples of cMWS coral (Mann-Whitney U test, W = 15, p = 0.012, n = 10). Healthy coral mucus had an average of 4.8×10^2^ (SE±176.83) CFU/ml, whereas diseased fragments had an average of 1.3×10^4^ (SE±4122.13) CFU/ml. Healthy coral also had a significantly lower proportion of *Vibrio* spp. as compared to diseased coral (Mann-Whitney U test, W = 15, p = 0.012, n = 10). Healthy coral had an average of 2.4% (SE±1.19%) *Vibrio* spp. compared to diseased coral, which had an average proportion of 28% (SE±5.35%). Both the abundance of bacteria and the percentage of cells that were *Vibrio* spp. were significantly higher in cMWS samples, suggesting similar responses of bacterial communities after the onset of disease on coral in the field and in infection trials.

### Re-isolation of Strain OCN002 from Diseased Coral in Infection Trials

If strain OCN002 was the cause of cMWS in the infection trials described above, it should be possible to re-isolate the strain from fragments of coral that showed signs of disease; re-isolation of a putative pathogen from subjects of an infection trial is one of Henle-Koch’s postulates for determination of disease causation [36], [37]. To facilitate identification of strain OCN002, PCR primers were designed to amplify a 1.2 kb region of DNA that includes a 594 bp open reading frame similar in sequence to the *moxR* genes of several bacteria and two flanking non-coding intergenic regions. The *moxR* gene is predicted to encode a AAA+ ATPase. This region was chosen based on the presence of two intergenic regions, which are not well conserved, and therefore served as a reasonable identifier of strain OCN002. All 70 of the bacterial isolates tested from the mucus of the seven coral fragments (ten from each coral) that showed signs of MWS after exposure to OCN002 yielded 1.2 kb PCR products with sequences identical to that from OCN002, suggesting that the recovered isolates were the same as, or very similar to, strain OCN002.

Sequence analysis suggests, but does not confirm, that the bacteria isolated from diseased coral were part of the clonal population added to the aquaria in the infection trials. To determine if direct descendants of OCN002 were recovered from diseased coral, a replicating plasmid was introduced to strain OCN002 to genetically tag the bacteria, and the resulting strain was used to infect fragments of *M. capitata*. All 30 bacterial isolates tested after recovery from laboratory infected corals contained the plasmid, pRL1383a, based on plasmid recovery and sequence analysis. In addition, each of the 30 isolates produced a PCR product with the same sequence as that of the *moxR* region of strain OCN002. Taken together, these results indicate that the specific strain of OCN002 containing plasmid pRL1383a, added during the infection trials, was recovered from the coral fragments that developed lesions. Conversely, no CFUs were recovered from coral exposed to OCN002 that remained healthy throughout the course of the infection trial on medium selective for the plasmid, suggesting that strain OCN002 does not persist on corals if it does not promote the disease state.

### Strain OCN002 is a Strain of *Vibrio owensii*

**Figure 4:**
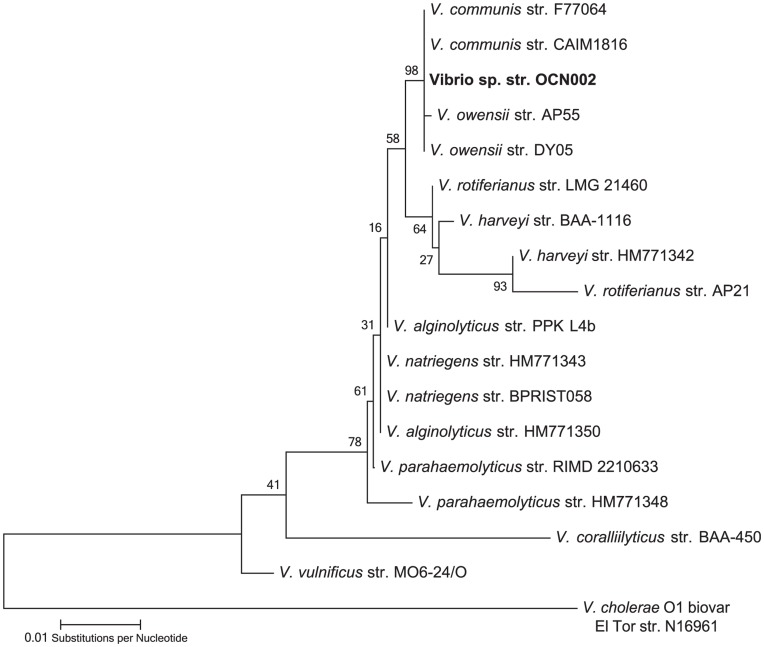
Phylogenetic tree showing relatedness among members of the Harveyi clade constructed using sequences of *Vibrio rrsh* genes, which encodes the 16S rRNA. The evolutionary history was inferred using the Maximum Likelihood method. The tree with the highest log likelihood is shown. The percentage of trees in which the associated taxa clustered together is shown next to each branch.

To classify OCN002 taxonomically, the sequence of its 16S ribosomal RNA (*rrsH*) gene was determined and analyzed. Using the universal primers 8F and 1513R, 1505 bp of the 1542 bp *rrsH* gene of strain OCN002 was amplified by PCR [38] and sequenced directly using the primers used for amplification. Nucleotide designations at each position were unambiguous, indicating that if multiple copies of the *rrsH* gene are present in this organism, they are likely exact copies of one another. BLAST analysis of the resulting 1324 bp sequence indicated that strain OCN002 is a member of the genus *Vibrio*, consistent with its ability to grow on TCBS medium. A bifurcating phylogenetic tree was generated using a region of the 16S rRNA genes from strain OCN002 and 17 other bacteria representing 9 species of *Vibrio* (Figure 4). Strain OCN002 appears to be closely related to multiple strains of *V. communis*, *V. owensii,* and *V. harveyi*, exhibiting greater than 99% identity in the sequence of the *rrsH* genes, indicating that OCN002 is in the monophyletic Harveyi clade [39].

Members of the Harveyi clade can be differentiated from one another by metabolic capabilities, a list of which is compiled in Table 1. Strain OCN002 is positive for both DNAse and extracellular protease activity and has γ-hemolytic activity. Characteristic swimming and swarming motility were observed, and the strain grew in liquid LB medium amended to NaCl concentrations of 0.1% to 6%. No growth was observed in LB at 10% NaCl or in the absence of added salt, but viable cells could be isolated after 4 days of incubation under the former conditions. Characteristic of bacteria in the Harveyi clade, strain OCN002 had an exceptionally fast growth rate. At 23°C, the average winter sea surface temperature of Kaneohe Bay [40], and 28°C, the average summer sea surface temperature of Kaneohe Bay [40], generation times during log phase growth were 9.9 and 8.0 min, respectively. OCN002 is unable to utilize trisodium citrate as a carbon source, a characteristic that separates *V. owensii* and *V. communis* from other members of the harveyi clade [41], [42]. In addition, the colony morphology of OCN002 was more similar to that of *V. owensii* than that of *V. communis.* On TCBS agar, OCN002 forms smooth, round colonies like *V. owensii* and *V. communis*. On other media types OCN002 forms opaque colonies like *V. owensii*, which differs from the translucent colonies of *V. communis* [41], [42]. Collectively, the physiological and morphological characteristics of OCN002 examined in this study are consistent with those of strains classified as *V. owensii*.

**Table 1:** Summary of metabolic and physiological characterization of *Vibrio owensii* strain OCN002, as compared to the type strains *V. owensii* strain DY05 [41] and *V. communis* strain R-40496 [42].

Test	*Vibrio owensii* strain OCN002	*Vibrio owensii* strain DY05	*Vibrio communis* strain R-40496
Nitrate Reduction to Nitrite	**+**	**+**	**+**
Indole production	**+**	**+**	**+**
Glucose fermentation	**+**	**+**	**+**
Arginine dihydrolase production	**−**	**−**	**−**
Urease production	**−**	**−**	**−**
β-glucosidase production	**+**	**ND**	**+**
Gelatinase production	**+**	**+**	**+**
β-galactosidase production	**+**	**−**	**+**
D-glucose assimilation	**+**	**+**	**+**
L-arabinose assimilation	**−**	**−**	**−**
D-mannose assimilation	**+**	**+**	**+**
D-mannitol assimilation	**−**	**+**	**+**
N-acetyl-glucosamine assimilation	**+**	**ND**	**+**
D-maltose assimilation	**+**	**ND**	**+**
Potassium gluconate assimilation	**+**	**+**	**ND**
Capric acid assimilation	**−**	**ND**	**ND**
Adipic acid assimilation	**−**	**ND**	**ND**
Malic acid assimilation	**+**	**+**	**ND**
Trisodium citrate assimilation	**−**	**−**	**−**
Phenylacetic acid assimilation	**-**	**ND**	**ND**
Growth at 23°C	**+**	**+**	**+**
Growth at 28°C	**+**	**+**	**+**
Growth at 37°C	**+**	**+**	**+**

(+) indicates a positive result and (−) indicates a negative result. ND indicates no data. API 20NE and indicator plate tests were quantified according to the manufacturer’s instructions, unless otherwise specified.

**Figure 5:**
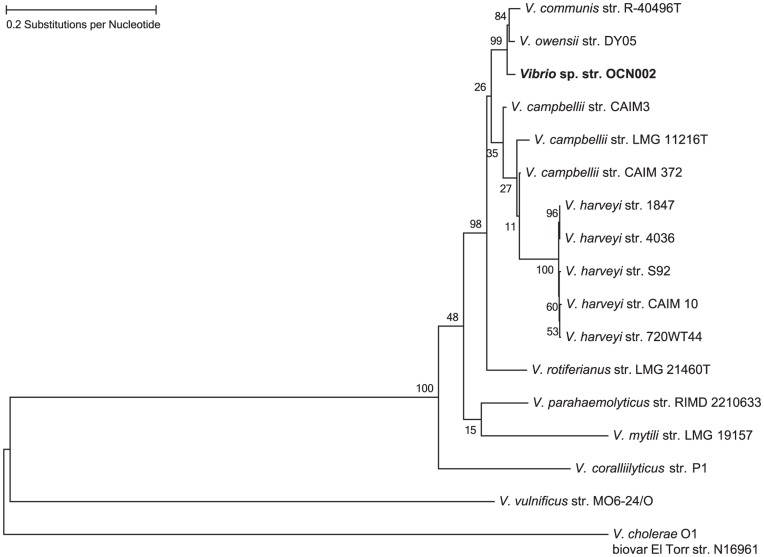
Phylogenetic tree showing relatedness of OCN002 constructed using *Vibrio* MLSA results. The evolutionary history was inferred by using the Maximum Likelihood method. The tree with the highest log likelihood is shown. The percentage of trees in which the associated taxa clustered together is shown next to each branch.

The genetic and metabolic similarities of strains within the Harveyi clade can complicate species designation of isolates. Recently, multilocus sequence analysis (MLSA) has been used to distinguish similar bacterial strains and categorize them phylogentically [43], [44]. Six housekeeping genes, *pyrH*, *gapA*, *mreB*, *ftsZ*, *gyrB*, and *topA*, were amplified by colony PCR from strain OCN002, sequenced, and concatamerized. The resulting 3098 bp sequence was aligned with corresponding sequences from 17 other strains representing 11 species of the genus *Vibrio*, and a maximum likelihood phylogenetic tree was generated (Figure 5). The concatemer alignment indicated that OCN002 is most closely related to the recently described species *V. owensii* [41]. Thus, from the combined characterization and MLSA of OCN002, we conclude that this bacterium is a strain of *V. owensii*.

## Discussion

Chronic *Montipora* white syndrome (cMWS) is a coral disease that has been killing the common reef coral, *Montipora capitata*, in Kāne?ohe Bay for the past several years [34]. We used a rapid screening method to initially identify a potential coral pathogen responsible for this disease and then subsequently isolated, identified and demonstrated that the bacterium, *V. owensii* strain OCN002, is a pathogen capable of causing cMWS in *M. capitata*. Identification of the etiological agent for this disease is an important first step towards future management and the development of conservation strategies for our dwindling reef resources.

The pathogen, *V. owensii*, is a recently described species of the Harveyi clade isolated from diseased crustaceans in Australia [41]. The Harveyi clade currently consists of 11 species: *V. harveyi, V. rotiferianus, V. alginolyticus, V. campbellii, V. parahaemolyticus, V. mytili, V. natriegens, V. azureus V. communis, V. jasicida* and *V. owensii*, many of which are associated with disease [24], [45]–[48]. The first three of these species along with *V. proteolyticus* have been implicated in causing Yellow Band Disease in corals from the Indo-Pacific and Caribbean [24]. YBD is caused by a consortium of bacteria; in infection trials, disease signs were only observed when coral were infected with multiple species concurrently [24]. In contrast, cMWS resulted from infection by strain OCN002 alone, suggesting that OCN002 was sufficient to cause disease. This is similar to other Indo-Pacific coral pathogens that cause tissue loss diseases. *V. shiloi* and *V. coralliilyticus* are temperature-dependent pathogens of the corals *Oculina patogonica* and *Pocillopora damicornis*, respectively [29], [32]. *V. coralliilyticus* has also been implicated in disease causation in acroporids and montiporids in other regions of the Indo-Pacific [25]. These species are distinct from the Harveyi clade; *V. shiloi* is related to the unclassified *V. mediterterranei* and the Splendidus clade, and *V. coralliilyticus* is the namesake of the Coralliilyticus clade [20], [29], [39]. *V. owensii* has also recently been isolated from *Acropora* white syndrome lesions in American Samoa, but its role as a coral pathogen there is yet to be determined [49]. The American Samoa studies indicate that *V. owensii* could be acting as a coral pathogen in other areas in the Pacific as well.

Disease states in infection trials were consistent with those in the field. Field and laboratory diseased fragments both had a higher abundance of culturable bacteria, particularly of the genus *Vibrio*. Infection by OCN002 under laboratory conditions had an average incubation period of 28 days, similar to the rate of cMWS transmission between fragments under laboratory conditions [34]. In the field, chronic MWS is a slow moving disease, killing colonies at a rate of approximately 3% of a colony per month [34]. In contrast, other coral tissue loss diseases progress far more quickly in the field and also have a shorter incubation period in infection trials [17], [21], [25].

In this study, it was conclusively demonstrated that bacteria re-isolated from diseased fragments infected in the laboratory were derived from the initial pure culture of OCN002 used to inoculate healthy corals. Coral specimens are brought in from the field with a natural bacterial flora, and *rrsH* gene sequencing is insufficient to differentiate between the resident bacterial flora and inocula used in infection trials. In addition to the *rrsH* gene sequence, the sequence of a second genomic region was compared between OCN002 and bacteria isolated from diseased coral. Inclusion of a sequence not expected to encode part of a protein or functional RNA molecule like the one used with OCN002 would be expected to increase the resolution. Ultimately, a plasmid capable of replication in strain OCN002 was used to genetically tag cells of OCN002 used in infection trials to provide conclusive evidence that strain OCN002 had been re-isolated from laboratory infected coral fragments. Use of the tagged strain also allowed the determination that OCN002 had not colonized the extracellular mucus of fragments that remained healthy, even those in the same aquarium as fragments that showed signs of MWS. Although it is technically possible that some bacteria lost the plasmid, a measurable curing rate for the plasmid was not observed under laboratory growth conditions.

The methods described in this study were developed to fulfill Henle-Koch’s four postulates of disease causation [36], [37]. Henle-Koch’s postulates are a set of criteria developed to establish a causal relationship between a potential pathogen and a disease [36], [50]. The isolation of OCN002 and subsequent growth in an axenic culture fulfilled Henle-Koch’s second postulate. Induction of disease anew in healthy laboratory specimens with OCN002 fulfilled the third postulate. Re-isolation of OCN002 from experimentally diseased specimens fulfilled the fourth postulate. Fulfillment of the first postulate, finding the pathogen in all diseased organisms, is likely to be difficult due to the limited range of observable responses a coral can have to a stressor, e.g., bleaching, cell lysis, growth anomalies, and discoloration, which results in the ambiguity of identified “white syndrome” diseases [51]–[53]. Hence, there may be different pathogens or stressors that are causing “white syndrome” for *M. capitata*. However, these postulates are thought of as guidelines, and for many diseases, such as typhoid fever, diphtheria, leprosy, and cholera, the accepted pathogens do not fulfill all the postulates [53], [54].

Currently, little is known of the virulence mechanisms used by strain OCN002 in the infection of *M. capitata*. However, three traits found in OCN002 (swimming motility, swarming motility and protease activity) have been shown to be important in the virulence of other bacteria. Swimming motility is typically dependent on polar flagella, which can mediate attachment to hosts [55]. In *V. coralliilyticus* expression of the *fhlA* gene, which encodes a membrane protein for transport of flagellar proteins to the cell surface, is important for pathogensis [56]. Swarming motility relies on lateral flagella, which has been extensively studied in *V. parahaemolyticus* and has since been shown in several other members of the Harveyi clade [57]–[59]. Similar to some members of the Harveyi clade, the growth rate of OCN002 was exceptionally fast, yet average time to infection was 28 days. In this study, the rate of pathogen growth does not correlate with time to infection or rate of disease progression [34]. In contrast, other bacterial pathogens of coral have been found to infect after a comparatively shorter incubation period in infection trials as well as progress rapidly in the field [25]. Lastly, extracellular protease activity has been suggested to contribute to the pathogenesis of *V. coralliilyticus* in its interaction with coral [32], [60].

The concentration of bacteria used in infection assays in this study and others is orders of magnitude higher than that expected in seawater for the bacterium of interest, suggesting that either infection relies on introduction of a high concentration of bacteria from a source other than the surrounding seawater, or infection results when some event, such as compromised host tissue, permits growth of bacteria to a sufficient concentration after introduction. These observations point to the possibility of a vector providing an environment to reach infectious doses and transfer among hosts. The coral pathogen *V. shiloi* uses the fireworm *Hermodice caranculata* as a winter reservoir [61], and human pathogens *V. cholerae* and *V. parahaemolyticus* attach to the chitin exoskeletons of copepods [62], [63]. Pathogen transfer and damage to host tissue could also be accomplished by coral predators, [64], [65]. Future research is planned to examine potential vectors associated with cMWS.

## Materials and Methods

### Growth of Bacteria

Marine bacteria were grown at 25°C in glycerol artificial seawater (GASW) medium [66] with the exception that Rila salts were replaced with Instant Ocean® (Spectrum Brands Inc., Atlanta, GA). Thiosulfate Citrate Bile Salts Sucrose (TCBS) solid medium was prepared according to the manufacturer’s instructions (BD, Franklin Lakes, NJ). *Escherichia coli* strains were grown in Luria-Bertani (LB) medium. Concentrations of antibiotics were 100 µg/ml for ampicillin, 50 µg/ml for kanamycin and 100 µg/ml for spectinomycin.

### Screen for a Potential MWS Pathogen

A fragment of *Montipora capitata* with cMWS from reefs surrounding Coconut Island in Kaneohe Bay was crushed, diluted 1∶10 in FSW, and spread on GASW plates in triplicate. From the 1710 colonies that grew on plates overnight, 50 were isolated, grown with aeration for approximately 15 hours, washed twice and resuspended in FSW, and inoculated in groups of 5 into aquaria (9.4 L) containing 3 healthy *M. capitata* fragments in FSW. Control tanks were inoculated with FSW only. Corals were held under ambient light and temperature, elevated on plastic grates to promote water flow, and visually monitored daily for signs of tissue loss. Aquaria air pumps provided water circulation, and water was changed every 4 days. Experiments were terminated following mortality in infection tanks. All necessary permits were obtained for the described field studies, including special activity permit number 2011-68 from the Hawaii Department of Land and Natural Resources for the collection of corals.

### Infection Trials with OCN002

From the initial screening, one bacterium, OCN002, was found to produce tissue loss. Additional infection trials with strain OCN002 were as described above with minor modifications. Four fragments instead of three were present in each aquarium. Each run consisted of a water control tank (no bacteria added), a bacterial control tank (inoculated with strain OCN004), and experimental tanks to which the test bacterium OCN002 was added. Cultures of inocula were grown to approximately 10^9^ cells/ml and final concentrations after inoculation were approximately 6×10^6^ cells/ml of tank water based on CFU produced on solid GASW. The inoculum was pipetted directly over each coral fragment, while care was taken to not cause physical damage to the fragment.

Control strain OCN004 was isolated from extracellular mucus of healthy *M. capitata* based on its ability to grow on GASW solid medium and classified as a member of the genus *Alteromonas* based on the sequence of the corresponding *rrsH* gene (Accession # JX152761). Coral fragments did not display signs of tissue loss when exposed to cultures of OCN004.

### Bacterial Abundances and Proportions of *Vibrios* in MWS Coral Fragments

Mucus was collected from diseased and healthy *M. capitata* colonies from experimental corals in infection trials (n = 13/treatment) as well as from the field (n = 5/condition) and assessed for numbers of CFUs on GASW and TCBS media, the latter of which is selective for the genus *Vibrio* [67]. Extracellular mucus was removed with a pipettor, vortexed, and plated either directly or diluted in FSW on GASW and TCBS solid media in triplicate. Colonies were counted after 24 hours. To determine changes during infection trials, CFUs per ml of mucus were determined immediately after collection from the field and subsequently upon completion of the infection trial.

### Conjugation of Plasmid pRL1383a and Re-isolation of Strain OCN002 Post-infection

To allow identification of strain OCN002 post-infection, the RSF1010-derived plasmid pRL1383a was conjugated into strain OCN002 to add a genetic tag to the strain [68]. Conjugation was by tri-parental mating with *E. coli* strains DH5α MCR with the self-transmissible plasmid pEVS104 or the mobilizable plasmid pRL1383a as previously described, with the exception that LB was used in place of heart infusion medium [69], [70]. Transconjugants were selected on LB with spectinomycin, resistance to which is encoded on pRL1383a, and ampicillin, to which strain OCN002 is naturally resistant.

Strain OCN002 containing pRL1383a was used in infection trials as described above. Mucus from coral was plated in triplicate on GASW, TCBS, and GASW with spectinomycin, and resulting colonies were counted and recorded. Plasmids were isolated from 10 colonies per plate as described previously [71]. The identity of pRL1383a was confirmed by PCR and subsequent sequencing of the product using the plasmid-specific primers pRL1383a MCS-F and pRL1383a MCS-R (Table S1).

### DNA Sequence Analysis of Strain OCN002

DNA primers used in this study are listed in Table S1. The *rrsH* gene of OCN002 was amplified using universal primers 8F and 1513R [72], sequenced with the same primers used for PCR (Accession #JX127215), and aligned using BioEdit [73] with 17 other sequences from species in the *V. harveyi* clade [39] and others in GenBank. A maximum likelihood tree was constructed using the generalized time-reversible (GTR) algorithm [74], and 1000 bootstrap replicates were performed using MEGA5 [75]. All positions containing gaps and missing data were eliminated.

For multilocus sequence analysis of OCN002, the *pyrH*, *gapA*, *mreB*, *ftsZ*, *gyrB*, and *topA* genes were amplified by PCR using primers described previously [39], sequenced (Accession #s JX127216, JX127217, JX127218, JX127219, JX127220, and JX127221, respectively), concatamerized [39], and aligned in BioEdit against corresponding concatamerized sequences from 16 other species of the Harveyi clade, Cholera clade, Vulnificus clade, and Coralliilyticus clade. Sequences were from NCBI and the open database resource <http://www.taxvibrio.lncc.br/> [43]. A maximum likelihood tree was constructed with 1000 bootstrap replicates using the concatemers in MEGA5 and the GTR algorithm as described above [75].

To establish an identifier of strain OCN002, genomic DNA from OCN002 was partially digested with Sau3A1 and 2 to 4 kb size-fractionated fragments were cloned into the BamHI site of plasmid pBluescript S/K+. A 2976 bp genomic DNA fragment was sequenced (Accession # JX127222) from one clone using M13 forward and reverse priming sites [76] on the plasmid initially and subsequently within the fragment using primers VPR4-Walk1R, VPR4-Walk2R, and VPR4-Walk3R. From this sequence a 1201 bp region flanked by primers UTR-MoxR-UTR-F and UTR-MoxR-UTR-R was chosen to be used as an identifier of strain OCN002.

### Metabolic Characterization of Strain OCN002

To aid in discrimination between *Vibrio* species, biochemical and metabolic tests were conducted. To test for extracellular protease, DNase, and hemolysin activity, OCN002 was streaked on Skim milk agar, Methyl green DNase agar, and 5% sheep blood TSA, respectively (BD, Franklin Lakes, NJ). Additional biochemical tests were performed with API 20NE strips (Biomériux Durham, NC) in triplicate with the provided AUX media and artificial seawater (GASW without glycerol, tryptone, or yeast extract). The indicator plates and API 20NE strips were incubated at 25°C for 24 hours and visually evaluated according to the manufacturer’s guidelines.

Salt tolerance was measured in LB supplemented with the following concentrations of NaCl: 0, 0.1, 0.5, 1.0, 3.0, 5.0, 6.0, and 10.0% (w/v). 2 ml cultures of OCN002 at each NaCl concentration were inoculated in triplicate from an overnight culture. Growth was assessed after 12 h incubation at 25°C by measuring the optical density of cultures at 600 nm.

Examination of the effect of temperature on growth employed triplicate 200 ml cultures in GASW inoculated from an overnight culture and incubated at 23°C or 28°C. Numbers of CFU from cultures plated every hour for 24 hours were used to calculate generation times.

### Statistical Analysis

Infection trial data was analyzed using McNemar’s test, a non-parametric method used for nominal data [77]. Changes in bacterial abundances and proportions of *Vibrio* species in infection trials were calculated as the difference between colony counts from the pre-trial fragments and the same fragments at the end of the infection trials. Differences in bacterial abundances within each group (control, control bacteria, pathogen exposed healthy, pathogen exposed diseased) were compared using a nonparametric Wilcoxon signed-rank test. The culturable bacterial abundances from field specimens and the between treatment changes in bacterial abundances, including the proportion of *Vibrios* for the laboratory specimens, were analyzed using a nonparametric Mann-Whitney U test.

## Supporting Information

Table S1 **Oligonucleotides used in this study.**
(DOC)

Table S2 **Summary of CFU counts and calculations for fragments used in infection trials.** Changes in bacterial abundance and proportion of *Vibrio* species were calculated as the difference between colony counts from the pre-trial fragments and corresponding fragments at the end of the infection trials. The pre and post-trial levels of culturable bacteria and proportion of *Vibrio* species were determined for each treatment. Seawater indicates fragments to which seawater was added as a control; OCN004 indicates fragments to which bacteria from healthy coral were added as a control, OCN002-healthy indicates fragments that remained healthy after addition of OCN002; and OCN002-diseased indicates fragments that developed tissue loss after addition of OCN002.
(DOC)
