# Supplementary material for: Vibrio owensii Induces the Tissue Loss Disease Montipora White Syndrome in the Hawaiian Reef Coral Montipora capitata
Source: PLoS One. 2012 Oct 8;7(10):e46717. doi: 10.1371/journal.pone.0046717 (PMC3466290; doi:10.1371/journal.pone.0046717)
Supplement: Table S1 — Oligonucleotides used in this study. (DOC) [file pone.0046717.s001.doc]

| **Primer Name** | **Primer Sequence** | **Description** | **Citation** |
| --- | --- | --- | --- |
| pRL1383a MCS-F | 5' CGAAGTTATATTCGATGCGG 3' | Forward primer for the non-transmissible vector pRL1383a | This study |
| pRL1383a MCS-R | 5' CATTATGGTGAAAGTTGGAACC 3' | Reverse primer for the non-transmissible vector pRL1383a | This study |
| 8F | 5' AGAGTTTGATCCTGGCTCAG 3' | Forward primer for the 16S rRNA gene | Aebischer et al. 2006 |
| 1513R | 5' GGTTACCTTTGTTACGACTT 3' | Reverse primer for the 16S rRNA gene | Aebischer et al. 2006 |
| VrecA130F | 5’ GTCTACCAATGGGTCGTATC 3’ | Forward primer for the Vibrio recA gene | Sawabe et al. 2011 |
| VrecA720R | 5’ GCCATTGTAGCTGTACCAAG 3’ | Reverse primer for the Vibrio recA gene | Sawabe et al. 2011 |
| VpyrH80F | 5’ GATCGTATGGCTCAAGAAG 3’ | Forward primer for the Vibrio pyrH gene | Sawabe et al. 2011 |
| VpyrH530R | 5’ TAGGCATTTTGTGGTCACG 3’ | Reverse primer for the Vibrio pyrH gene | Sawabe et al. 2011 |
| VgapA150F | 5’ AACTCACGGTCGTTTCAAC 3’ | Forward primer for the Vibrio gapA gene | Sawabe et al. 2011 |
| VgapA899R | 5’ CGTTGTCGTACCAAGATAC 3’ | Reverse primer for the Vibrio gapA gene | Sawabe et al. 2011 |
| VmreB12F | 5’ ACTTCGTGGCATGTTTTC 3’ | Forward primer for the Vibrio mreB gene | Sawabe et al. 2011 |
| VmreB999R | 5’ CCGTGCATATCGATCATTTC 3’ | Reverse primer for the Vibrio mreB gene | Sawabe et al. 2011 |
| VftsZ75F | 5’ GCTGTTGAACACATGGTACG 3’ | Forward primer for the Vibrio ftsZ gene | Sawabe et al. 2011 |
| VftsZ800R | 5’ GCACCAGCAAGATCGATATC 3’ | Reverse primer for the Vibrio ftsZ gene | Sawabe et al. 2011 |
| VgyrB274F | 5’ GAAGTTATCATGACGGTACTTC 3’ | Forward primer for the Vibrio gyrB gene | Sawabe et al. 2011 |
| VgyrB1171R | 5’ CCTTTACGACGAGTCATTTC 3’ | Reverse primer for the Vibrio gyrB gene | Sawabe et al. 2011 |
| VtopA400F | 5’ GAGATCATCGGTGGTGATG 3’ | Forward primer for the Vibrio topA gene | Sawabe et al. 2011 |
| VtopA1200R | 5’ GAAGGACGAATCGCTTCGTG 3’ | Reverse primer for the Vibrio topA gene | Sawabe et al. 2011 |
| M13-F | 5’ GTAAAACGACGGCCAGTG 3’ | Forward primer for the M13 region for vector pBluescript SK(+) used in this study | Messing 1983 |
| M13-R | 5’ GGAAACAGCTATGACCATG 3’ | Reverse primer for the M13 region for vector pBluescript SK(+) used in this study | Messing 1983 |
| VPR4-Walk1R | 5’ CCCTCCTGCTTATCCGATTTC 3’ | Internal primer developed for sequencing of cloned section of OCN002 genome | This study |
| VPR4-Walk2R | 5’ CTGAATACGACCAAAGCTGG 3’ | Internal primer developed for sequencing of cloned section of OCN002 genome | This study |
| VPR4-Walk3R | 5’ GACTCTGCCAAGATCGTTGT 3’ | Internal primer developed for sequencing of cloned section of OCN002 genome | This study |
| UTR-MoxR-UTR-F | 5’ GTAATCGCAATACAAGCGAGGGTCG 3’ | Forward primer developed from OCN002 chromosomal DNA | This study |
| UTR-MoxR-UTR-R | 5’ GACCATTCGCTGCCAATTCTGGTTG 3’ | Reverse primer developed from OCN002 chromosomal DNA | This study |

Aebischer T, Fischer A, Walduck A, Schlötelburg C, Lindig M, et al. (2006) Vaccination prevents Helicobacter pylori-induced alterations of the gastric flora in mice. *FEMS Immunol Med Mic* 46: 221–229.

Messing J. (1983). New M 13 vectors for cloning. *Method Enzymol* 101:20-78.

*Sawabe T, Kita-Tsukamoto K, Thompson FL. (2007). Inferring the evolutionary history of Vibrios by means of multilocus sequence analysis.* J Bacteriol *189:7932–7936.*
