# Supplementary material for: Vibrio owensii Induces the Tissue Loss Disease Montipora White Syndrome in the Hawaiian Reef Coral Montipora capitata
Source: PLoS One. 2012 Oct 8;7(10):e46717. doi: 10.1371/journal.pone.0046717 (PMC3466290; doi:10.1371/journal.pone.0046717)
Supplement: Table S2 — Summary of CFU counts and calculations for fragments used in infection trials. Changes in bacterial abundance and proportion of Vibrio species were calculated as the difference between colony counts from the pre-trial fragments and corresponding fragments at the end of the infection trials. The pre and post-trial levels of culturable bacteria and proportion of Vibrio species were determined for each treatment. Seawater indicates fragments to which seawater was added as a control; OCN004 indicates fragments to which bacteria from healthy coral were added as a control, OCN002-healthy indicates fragments that remained healthy after addition of OCN002; and OCN002-diseased indicates fragments that developed tissue loss after addition of OCN002. (DOC) [file pone.0046717.s002.doc]

| Treatment | *N* | Pre-trial GASW Average CFUs per ml of mucus | Average GASW CFUs per ml of mucus | Average Change (CFUs/ml) | Test Statistic (W) | Wilcoxon-signed rank test (p-value) | Pre-trial TCBS CFUs per ml of mucus | Average TCBS CFUs per ml of mucus | Average proportion *Vibrios* | Percent Change in *Vibrio* | Test Statistic (W) | Wilcoxon-signed rank test (p-value) |
| --- | --- | --- | --- | --- | --- | --- | --- | --- | --- | --- | --- | --- |
| Seawater | 13 | 13.66 | 2.72 | -1.0 X 10^1^ (SE±3.79) | 43 | 0.07 | 3.63 | 0.05 | 1.84% | -14.0% (SE±8.07%) | 36 | 0.01 |
| OCN004 | 13 | 13.66 | 1.26 | -1.1 X 10^1^ (SE±4.52) | 67 | 0.01 | 3.63 | <0.01 | <1% | -16.0% (SE±7.72%) | 36 | 0.01 |
| OCN002- healthy | 7 | 13.66 | 9.57 | +1.3 X 10^1^ (SE±466.98) | 9 | 0.06 | 3.63 | 0.02 | 0.21% | -3.0% (SE±0.36%) | 3 | 0.051 |
| OCN002- diseased | 6 | 13.66 | 2390.60 | +2.5 X 10^3^ (SE±9.55) | 28 | 0.01 | 3.63 | 2203.57 | 92.18% | +78.0% (SE±26.35%) | 28 | 0.01 |
